# A randomized trial to evaluate a modified tracheal catheter with upper and lower balloons for anesthetic administration: effect on the cardiovascular, stress response, and comfort in patients undergoing laparoscopic cholecystectomy

**DOI:** 10.1186/s12871-019-0883-7

**Published:** 2019-11-15

**Authors:** Yuenong Zhang, Zhiwen Zeng, Guangwen Xiao, Weiqiang Zhang, Weixiong Lin, Jingdan Deng

**Affiliations:** 1 First Department of Anesthesiology, People’s Hospital of Meizhou City, Meizhou, Guangdong Province China; 2 Department of Laboratory Medicine, Jiaying University of Meizhou City, Meizhou, Guangdong Province China

**Keywords:** Induction of anesthesia, Tracheal intubation, Tracheal catheter with dual administration channels, Cardiovascular response, Comfort

## Abstract

**Background:**

We aimed to evaluate a modified endotracheal tube containing upper and lower balloons for anesthetic administration among patients undergoing laparoscopic cholecystectomy.

**Methods:**

Ninety patients scheduled to undergo laparoscopic cholecystectomy were randomly allocated to 3 equal groups: group A (conventional tracheal intubation without endotracheal anesthesia); B (conventional tracheal intubation with endotracheal anesthesia); and C (tracheal intubation using a modified catheter under study). Blood pressure, heart rate, angiotensin II level, blood glucose level, airway pressure before anesthesia (T1) were measured immediately after intubation (T2), 5 min after intubation (T3), and immediately after extubation (T4). The post-extubation pain experienced was evaluated using the Wong-Baker Face Pain scale. Adverse reactions within 30 min after extubation were recorded.

**Results:**

Systolic blood pressure, diastolic blood pressure, angiotensin II, and blood sugar level in group C at T2, T3 and T4, and heart rate at T2 and T4 were significantly lower than those in group A (*P* < 0.05); systolic blood pressure and blood sugar at T4, and angiotensin II levels at T2, T3, and T4 were significantly lower than those in group B (*P* < 0.05). Patients in group C reported the lowest post-extubation pain (*P* < 0.05 vs. Group A), and the lowest incidence of adverse events such as nausea, vomiting, and sore throat than that in groups A and B (*P* < 0.05).

**Conclusion:**

The modified endotracheal anesthesia tube under study is effective in reducing cardiovascular and tracheal stress response, and increasing patient comfort, without inducing an increase in airway resistance.

**Trial registration:**

The clinical trial was retrospectively registered at the Chinese Clinical Trial Registry with the Registration Number ChiCTR1900020832 at January 20th 2019.

## Background

Laparoscopy is widely applied for various types of abdominal surgeries, like cholecystectomy, as a minimally invasive procedure associated with less pain and faster recovery [1, 2]. The procedure requires insufflation of the abdomen with carbon dioxide for better visual access which increases the abdominal pressure. The increased intra-abdominal pressure during pneumoperitoneum is liable to decrease cardiac venous return, which results in elevated blood pressure and heart rate, causing adverse effects to the patients [3]. Therefore, improved management of general anesthesia in order to reduce complications is one of the keys to successful surgery [4].

Stimulation of the laryngeal structure by direct laryngoscopy during insertion of the endotracheal tube and tracheal stimulation during intubation induces a transient but intense cardiovascular stress in patients [5]. This discomfort can be significantly reduced by endotracheal anesthesia using local anesthetics such as lidocaine aerosol or gel prior to insertion of the endotracheal tube [6, 7]. Aerosol anesthetics, however, can be administered only once prior to the insertion of the endotracheal tube, which limits the duration of its effect. Also, potential concentration of dense drug particles on the inner wall of the catheter delays the onset of action. Similar challenges are encountered with the use of anesthetic gel with respect to inaccurate administration and insufficient maintenance time.

**Fig. 1:**
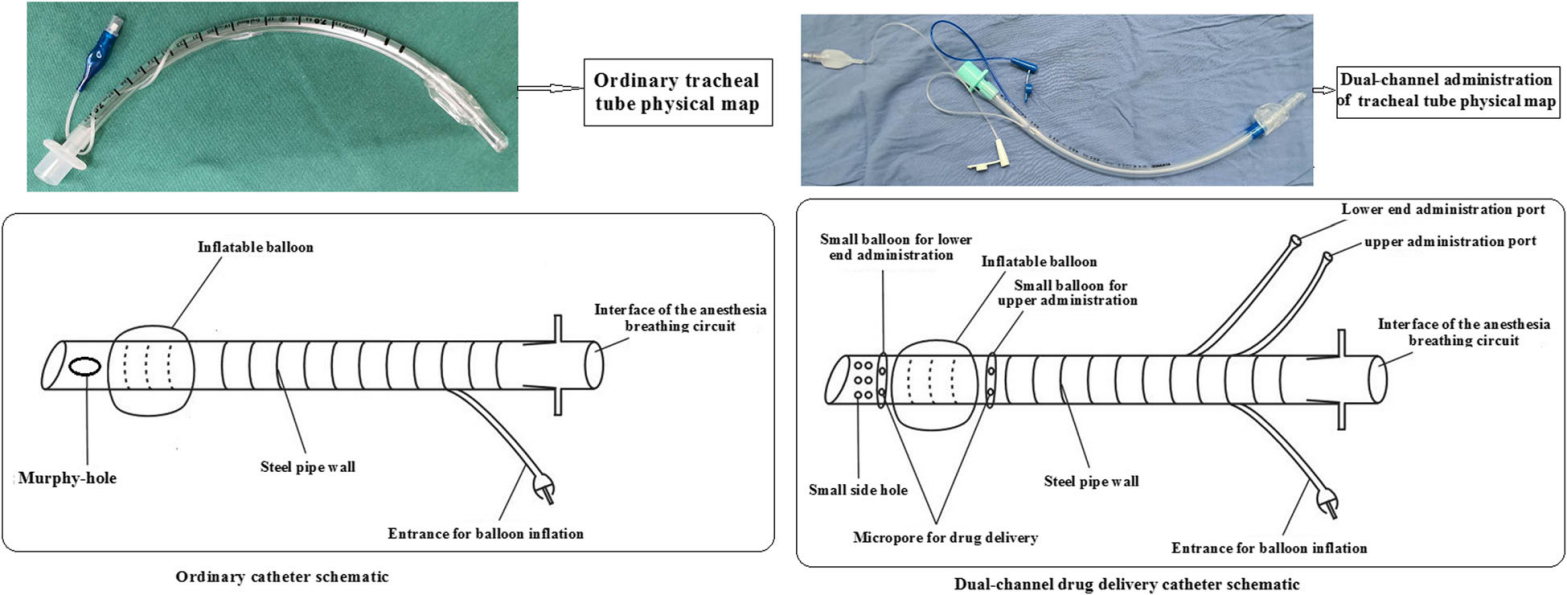
Comparison of the conventional catheter and dual-channel catheter

Taking cognizance of the above-listed pros and cons, a modified intratracheal catheter has been designed by our hospital, which contains upper and lower channels for more even anesthetic administration (Fig. 1). In the present study, we tested the modified catheter. We intended to demonstrate that the design modification involving addition of upper and lower drug dispensing chambers and replacement of the Murphy eye with multiple small miniholes can reduce the cardiovascular stress response associated with tracheal intubation.

## Methods

### General materials

CONSORT guideline has been followed for this study, and ethical approval has been granted by the Ethics Committee of People’s Hospital of Meizhou City, Guangdong Province, China. Written informed consent was obtained from all subjects prior to their enrolment. The clinical trial was retrospectively registered at the Chinese Clinical Trial Registry (http://www.chictr.org.cn/index.aspx) with the Registration Number ChiCTR1900020832 at January 20th 2019.

**Fig. 2:**
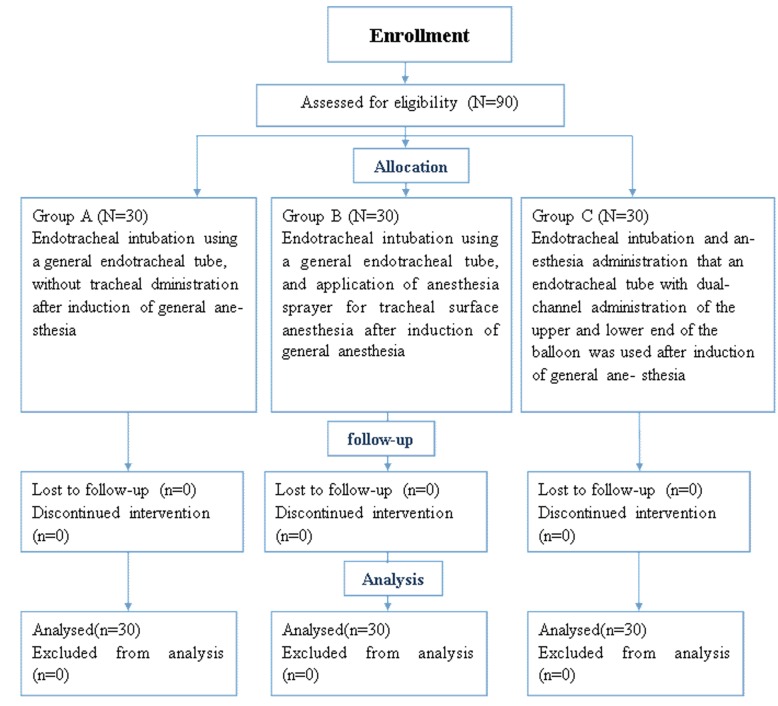
Schematic illustration of the randomized trial design, including enrollment, intervention allocation, and analysis

Ninety patients scheduled for cholecystectomy at our hospital between October 2017 and March 2019 were recruited by the study nurse for the prospective randomized double-blind clinical study. The inclusion criteria included: (1) patients aged > 25 years old; (2) patients diagnosed based on the “Diagnosis and Treatment Guidelines for Acute Biliary Infection” (2011 edition) released by the Surgery Branch of the Chinese Medical Association, (3) patients were scheduled for laparoscopic cholecystectomy under general anesthesia; and (4) patients classified as ASA (American Society of Anesthesiologists) I or II degree. The exclusion criteria included: (1) patients aged < 25 years or > 87 years; (2) patients were classified as Grade III or IV according to New York Heart Association (NYHA), or those with significant diseases such as hypertension, diabetes, lung disease, mental illness, or allergy to anesthetics; (3) patients had conditions that are known to cause difficulty in intubation; (4) patients were unable to understand or follow instructions normally. Especially, patients with lung disease were excluded, considering that such patients may secrete more sputum, causing blockage of block micropores. At the end of the screening period, a total of 90 patients successfully completed the study (Fig. 2).

Patients were randomly allocated to 3 equal groups using random numbers generated using Microsoft Excel by an investigator who was blinded to the medical situation of the subjects: group A (conventional tracheal intubation without endotracheal anesthesia); group B (conventional tracheal intubation with tracheal surface anesthesia); and group C (tracheal intubation using modified catheter under study). Each group was comprised of 30 patients. The sealed envelope containing the patient allocation information was under the custody of the research supervisor. After patients had signed the informed consent form, the investigator opened the sealed envelope to determine the allocated group. Throughout the study, the investigator and study nurse who performed the assessment, care providers, and participants remained blinded to the group assignment.

### Modified catheter: an intratracheal tube containing upper and lower balloons for anesthetic administration and miniholes for drug particle dispersion

Based on the commonly used intratracheal catheter, we modified its structure by adding the upper and lower balloons for anesthetic administration, and replacing the Murphy eye at the dispersion chamber with multiple miniholes. The two balloons at either end of the gas balloon can be controlled independently to spray out the anesthetic agent for wide and even distribution. The microholes on the surface produced using laser drill provide improved ventilation of the airway. The embedded three-way valve enables single or simultaneous administration.

### Anesthesia approach

All subjects were fasted for 8 h with no preoperative administration before surgery. The subjects were placed in the supine position to establish venous access and were connected to ECG monitor for continuous monitoring of heart rate, blood pressure, oxygen saturation, and entropy index. Prior to the induction of anesthesia (T1), blood pressure and heart rate were recorded and venous blood samples were collected. Intravenous anesthesia was induced using atropine 0.2 mg, etomidate 0.4 mg/kg, sufentanil 0.4 μg/kg, and atracurium 0.6 mg/kg, followed by maintenance with sevoflurane 1–2% and remifentanil 0.15–0.3 μg/kg/min. In addition, sufentanil 0.2 μg/kg was added prior to the start of the operation, and sufentanil 0.2 μg/kg was administered 15 min after the operation. Atracurium was administrated continuously during the operation to maintain muscle relaxation. Intratracheal surface anesthesia was performed differently for the three groups. For patients in group A, routine tracheal intubation was performed without tracheal administration. Patients in group B received 4 mL anesthesia spray (2% lidocaine) after anesthesia induction to achieve intratracheal surface anesthesia, followed by routine tracheal intubation. For patients in group C, 2 mL anesthesia spray (2% lidocaine) was administered from the lower channel connected to the administration balloon after anesthesia induction for intratracheal surface anesthesia; 5 s later, when intubation had reached the expected depth, 2 mL anesthesia spray (2% lidocaine) was administered from the upper channel; 15 min before completion of surgery, 2 mL 2% lidocaine was administered from the upper and lower channels, respectively. All intubations were performed within 90 s. After successful intubation, patients were connected to the breathing circuit of the anesthesia machine, and the time of connection was noted as T2 (immediately after endotracheal intubation). Parameters such as blood pressure and heart rate were collected and venous blood samples drawn by anesthesia assistant. All patients were ventilated with a tidal volume of 8 mL/kg, an inspiratory: expiratory (I:E) ratio of 1:2, and a respiratory rate of 12–14 breaths/min in 100% oxygen without positive end-expiratory pressure (PEEP), maintaining PetCO2 value of 35–45 mmHg. The entropy was maintained in the range of 40–60 to ensure adequate depth of anesthesia. Blood pressure and heart rate were measured 5 min after intubation (T3, before pneumoperitoneum), and the venous blood sample was collected as well. Carbon dioxide was insufflated into the peritoneal cavity up to a pressure of 12 mmHg. During the surgery, the patient was laid head-up and turned left. At the end of the surgery, pneumoperitoneum was evacuated and patients were kept in a supine position. Muscle relaxant antagonists atropine 0.3 mg and neostigmine 1 mg were administered after patients had regained spontaneous breathing. The intubation was removed after monitoring breathing rate (> 12 times per min), tidal volume (> 6 mL/kg), and SpO2 > 96%, and oxygen support was stopped 5 min later. The time point immediately after extubation was referred to as T4. Parameters including blood pressure and heart rate were collected and a venous blood sample was drawn. The time duration and time of extubation were recorded. The patient was then transferred to the anesthesia recovery room for observation.

### Parameter observation

Systolic blood pressure (SBP), diastolic blood pressure (DBP), and heart rate (HR) were recorded at each time point (from T1 to T4). Parameters reflecting airway pressure before, during and after pneumoperitoneum were recorded. Angiotensin II and blood glucose levels were measured with venous blood samples collected at each time point. The pain felt after extubation was scored by anesthesia assistants using the Wong-Baker Face Pain scale. The FACES scale is used to rate pain on a scale of 0–5, where 0 corresponds to no pain (“No Hurt”; illustrated as a face with a broad smile] and 5 corresponds to severe pain (“Hurts worst”; illustrated with a face with a frown and tears) [8]. Adverse reactions within 30 min after extubation such as nausea, vomiting, dizziness, sore throat, difficulty breathing, and low blood pressure were recorded.

### Statistical analysis

Data processing and analysis were performed using SPSS 21.0 software. Normally distributed continuous variables are expressed as mean ± standard deviation and between-group differences assessed using one-way ANOVA and independent t-test. The count data were analyzed using a Chi-squared test. The difference was statistically significant at *P* < 0.05.

## Results

### Demographic characteristics

Patients in the three groups showed no significant difference with respect to age, gender, body weight, and ASA status or with respect to the duration of surgery and time of extubation (all *P* > 0.05) (Table 1). The operation was performed to subjects from October 20, 2017, to March 14, 2019, and the observation of the last patient was completed on March 14, 2019.

**Table 1:** Intubation and extubation time of patients in the three groups

Characteristics	Group			F/ Chi-square value	*P*-value
	A (*n* = 30)	B (*n* = 30)	C (*n* = 30)		
Age (years)	57.4 ± 13.4	53.4 ± 15.9	55.4 ± 12.2	0.618	0.541
Gender (male/female)	16/14	17/13	15/15	0.268	0.875
Body weight (kg)	58.7 ± 11.2	61.9 ± 9.4	60.9 ± 10.1	0.755	0.473
ASA Classification (I/II)	12/18	13/17	11/19	0.278	0.870
Operation time (min)	60.3 ± 13.3	57.4 ± 16.9	58.3 ± 14.7	0.293	0.747
Extubation time (min)	10.4 ± 2.9	9.9 ± 3.6	9.6 ± 3.3	0.487	0.616

*ASA* American Society of Anesthesiology

### Comparison of SBP, DBP, heart rate, angiotensin II, and blood glucose at respective time points

**Fig. 3:**
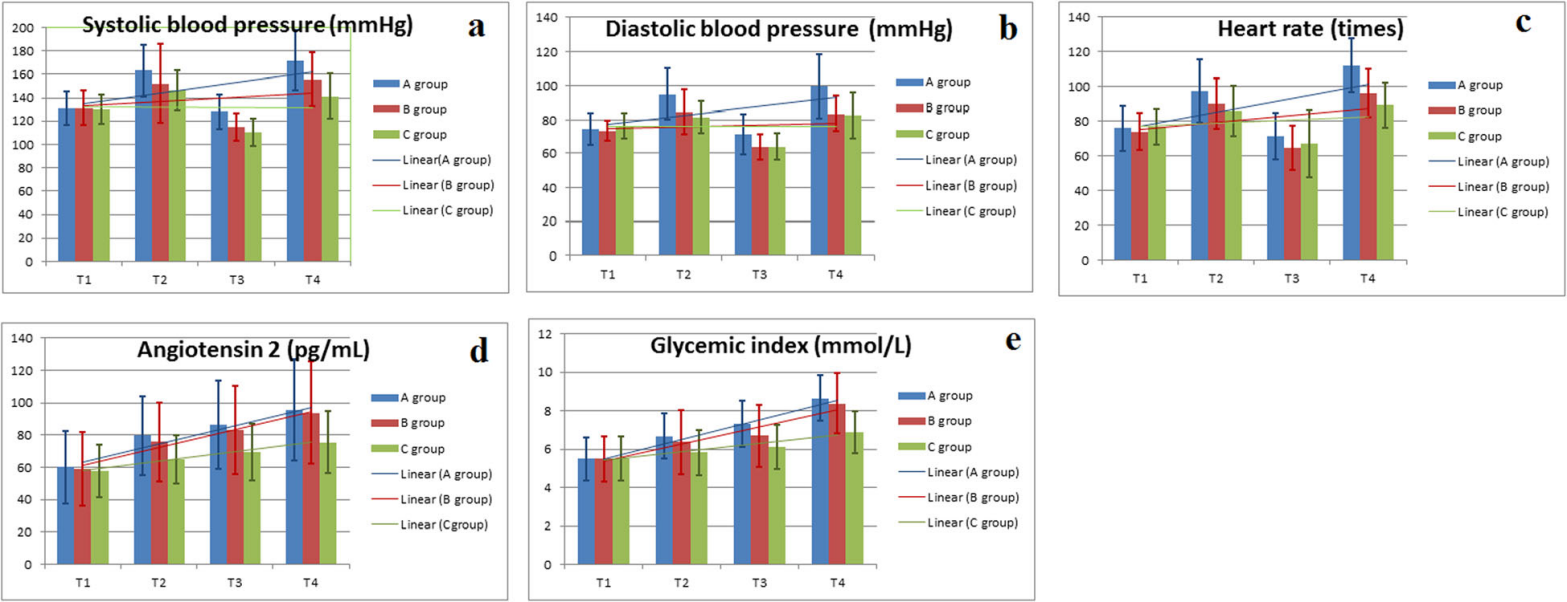
Comparison of systolic blood pressure (**a**), diastolic blood pressure (**b**), heart rate (**c**), angiotensin II (**d**), and glycemic index (**e**) at T1–T4 time points between the three groups

Fluctuations of SBP, DBP, and heartbeat were observed from T1 to T4 in all three groups. A common trend was that blood pressure increased from T1 to T2, decreased from T2 to T3, and subsequently increased again from T3 to T4. On comparing the trend of SBP and DBP, group C showed the slowest change, while group A showed the most significant fluctuation. On comparing the trend of DBP from T1 and T4, the changes in groups C and B were smooth compared with group A which showed a more remarkable change (Fig. 3a, b, c). The mean level of angiotensin II and mean blood glucose in the three groups showed a trend of gradual increase from T1 to T4. Similarly, patients in group C experienced a smooth and gradual increase (Fig. 3d, e). On comparing groups A and B, a significant difference was detected between SBP at T3 and T4, DBP at T2, T3, and T4, and heart rate at T4 (*P* < 0.05 for all). On comparing groups B and C, a significant difference was observed with respect to SBP at T4, angiotensin II level at T2, T3, and T4, and blood sugar at T4 (*P* < 0.05). On comparing between groups A and C, significant differences were observed with respect to SBP, DBP, angiotensin II at T2, T3 and T4, and heart rate at T2 and T4 (both *P* < 0.05) (Table 2).

**Table 2:** Pair-wise comparisons of SBP, DBP, heart rate, blood glucose, and angiotensin II levels at various time-points in the three groups

Characteristics	Comparison among groups	Statistical value	Time-points			
			T1	T2	T3	T4
Systolic blood pressure (mmHg)	A and B	*t* -value	0.093	1.679	2.511	2.853
		*P*-value	0.926	0.099	0.015	0.006
	B and C	*t*-value	0.129	0.458	1.482	2.506
		*P*-value	0.898	0.649	0.144	0.015
	A and C	*t* -value	0.238	2.987	3.432	5.238
		*P*-value	0.813	0.004	0.001	0.000
Diastolic blood pressure (mmHg)	A and B	*t* -value	0.489	2.968	2.811	4.179
		*P*-value	0.626	0.004	0.007	0.000
	B and C	*t* -value	−1.595	0.764	0.081	0.210
		*P*-value	0.116	0.448	0.936	0.835
	A and C	*t* -value	−0.831	3.907	2.796	4.022
		*P*-value	0.409	0.000	0.007	0.000
Heart rate (beats per min)	A and B	*t* -value	0.170	1.652	1.308	3.313
		*P*-value	0.866	0.104	0.196	0.002
	B and C	*t* -value	−0.112	1.248	0.273	1.611
		*P*-value	0.911	0.217	0.768	0.113
	A and C	*t* -value	0.075	2.651	1.651	5.041
		*P*-value	0.940	0.010	0.104	0.000
Angiotensin II (pg/mL)	A and B	*t* -value	0.147	0.329	1.130	0.969
		*P*-value	0.884	0.743	0.263	0.337
	B and C	*t* -value	0.257	2.104	2.086	2.686
		*P*-value	0.798	0.040	0.041	0.009
	A and C	*t* -value	0.430	2.575	3.705	3.447
		*P*-value	0.669	0.013	0.000	0.001
Glycemic index (mmol/L)	A and B	*t* -value	−0.044	0.830	1.766	0.769
		*P*-value	0.965	0.410	0.083	0.445
	B and C	*t* -value	−0.055	1.468	1.602	4.284
		*P*-value	0.957	0.148	0.115	0.000
	A and C	*t* -value	−0.101	2.858	4.014	5.971
		*P*-value	0.920	0.006	0.000	0.000

*SBP* Systolic blood pressure, *DBP* Diastolic blood pressure

### Airway resistance before, during, and after pneumoperitoneum

No significant between-group difference was observed with respect to the airway resistance before, during, or after pneumoperitoneum.

### Evaluation of pain with Wong-baker FACES pain rating scale

The severity of discomfort was evaluated using the Wong-Baker FACES Pain Rating Scale immediately after extubation. As shown in Table 3, patients in group C scored the lowest points (1.8 ± 1.69), which were significantly lower than that in group A (3.27 ± 2.85, *P* < 0.05); however, the scores in groups A and B were comparable.

**Table 3:** Wong-Baker FACES pain rating scale scores immediately after extubation in the three groups

		Group		
		A (*n* = 30)	B (*n* = 30)	C (*n* = 30)
Wong Baker FACES pain rating scale score	Mean	3.27	2.67	1.8^ab^
	SD	2.85	2.37	1.69
	Percentiles (25)	0	0	0
	Percentiles (50)	2	2	0
	Percentiles (75)	6	4	2
	Minimum	0	0	0
	Maximum	10	10	6

Compared with group A, ^a^*t* = 5.873, ^a^*P* = 0.019; Compared with group B, ^b^*t* = 2.662, ^b^*P* = 0.108

*SD* Standard deviation

### Adverse events

Incidence of nausea and vomiting in group A (40%) was significantly greater than that in groups B (16.7%) and C (10%). Vertigo was reported by 30% patients in group C, as against 13.3 and 23.3% patients in groups A and B, respectively; however, the between-group difference in this respect was not statistically significant. Notably, the incidence of sore throat in group C (6.7%) was significantly lower than that in group A (46.7%) and group B (26.7%). None of the subjects reported dyspnea or hypotension.

## Discussion

In the present study, we evaluated the safety and efficacy of the new intratracheal catheter in patients undergoing laparoscopic cholecystectomy. Tracheal intubation under general anesthesia is known to stimulate the renin-angiotensin system, which increases the level of angiotensin II. Therefore, the concentration of angiotensin II was used as a specific indicator of the intubation-induced stress response. Moreover, glycogenolysis and gluconeogenesis is upregulated in this setting, which induces an increase in blood glucose level. Angiotensin II has been used as a parameter reflecting hemodynamic variation during tracheal intubation in published literature [9–11]. Therefore, the level of angiotensin II and blood glucose were measured as quantitative parameters of the degree of irritation caused by endotracheal intubation. Our data indicates the safety and performance of the modified design in the studied patient group.

During endotracheal intubation, insertion of tracheal catheters and laryngoscope induces neural and chemical responses (including endocrine secretions) [12], followed by sympathetic nerve excitability. Tracheal intubation can increase sympathetic activity, which may induce dramatic changes in blood pressure [13]; this necessitates the use of anesthetic drugs or vasoactive drugs for hemodynamic stabilization. However, the residual effects of these drugs post-extubation often result in adverse effects. The dilemma pertaining to the administration of anesthetic drugs during intubation is a real challenge during anesthesia management [14].

One of the purposes of inhalational anesthesia management is to alleviate the cardiovascular response [12]. However, given the design of the conventional endotracheal tube, local anesthesia can only be applied once upon intubation [15]. In recent years, different types of endotracheal tubes have been designed which enable free intratracheal administration, for example, the endotracheal tube supporting one-way administration described by Wu ZH, et al. [16] and endotracheal tube supporting upper and lower anesthetic administration described by Zhao LQ, et al. [17]. Lidocaine administration via a single-channel tracheal tube was shown to effectively stabilize the circulation and shorten the weaning period and ICU stay [16]. Dual-channel anesthesia administration provides better hemodynamic control during intubation and extubation; however, with one-channel administration, the drug particles act only on a limited area of intratracheal surface owing to the unidirectional spray [17]. Still, the downside of dual-channel administration is that anesthesia occurs at the level of glottal closure. This may cause throat and glottis anesthesia, which may disable the protective reflexes. In addition, the residual anesthetic solution on the glottis may increase the risk of aspiration.

In this study, we tested a modified endotracheal tube intended to provide upper-and-lower administration; the tube allows for local anesthesia spray from balloons connected to the upper and lower ends, either separately or jointly. In addition, the drug solution is sprayed through microholes, which ensures adequate contact between the drug solution and the tracheal wall. Considering the extended length of the drug-delivery catheter, the single Murphy eye design was replaced with overlaid microholes to minimize the stimulation of the tracheal carina.

SBP, DBP, angiotensin II, and blood sugar (T2, T3, and T4), and heart rate (T2, T4) in group C were significantly lower than that in group A. The trend in the change of the respective parameters in group C from T1 to T4 was more gradual than that in group A. Both findings suggest that lidocaine sprayed in the endotracheal tube attenuates the airway-circulatory reflexes during emergence and extubation in patients receiving laparoscopic cholecystectomy. We further analyzed whether the effect was attributable to lidocaine itself or to the modified approach of administration by comparing the parameters between the groups B and C. The SBP (T4), angiotensin II (T2, T3, T4), and blood sugar (T4) in Group C were significantly lower than that in Group B. Similarly, the trend of change from T1 to T4 showed more gradual fluctuation in group C than group B. These results suggested that the modified intratracheal drug delivery catheter can further improve the airway-circulatory reflexes. However, the small sample size of our study should be considered while interpreting the results: despite the statistically significant analysis results, whether the differences between group B and C are clinically important should be further studied. Administration of atropine and neostigmine at the end of surgery may make the post-operative hemodynamic data less convincing. Yet according to the study design, atropine and neostigmine were provided at the end of surgery at the same dosage and the same time-point to all the included patients. This procedure is also part of the routine operative protocol at our hospital. Therefore, parallel comparison among the three groups of patients was feasible since the effects of the same post-operative medical treatment were experienced identically in all patients. The flexible control of anesthetic administration enabled by our device facilitated better control of sympathetic excitation and hemodynamics and helped achieve adequate tracheal surface anesthesia [18]. Moreover, a previous study demonstrated the efficiency of local lidocaine in causing relaxation of tracheal smooth muscle [19].

Sore throat is one of the most common adverse effects of tracheal intubation and general anesthesia (incidence rate: 33–44%) [20]. Causes of sore throat include cough due to unstable anesthesia, neck overextension, and excessive air pressure of airway or airbag, which causes damage to the airway mucosa and leads to edema in the glottic area [21]. Studies have shown that the use of a topical anesthetic ointment to lubricate the end of the tracheal tube before intubation or administration of anesthetic analgesics before and after surgery can alleviate postoperative sore throat symptoms [19, 20, 22]. However, despite the short-term efficiency, patient’s symptoms of sore throat tend to get worse about 2 h after surgery; in addition, throat edema and airway infection may even cause suffocation. The adverse events were evaluated using Wong-baker faces pain rating scale immediately after extubation. Patients in group C showed significantly lower scores than that in group A; Moreover, the incidence of nausea, vomiting, and sore throat was also significantly lower in group C (Table 4). Collectively, these results suggest the clinical benefits of the use of a modified intratracheal drug delivery catheter [23].

**Table 4:** Incidence of adverse effects within 30 min post-endotracheal extubation in the three groups

Group	Nausea and vomiting	Dizziness	Pharyngalgia	Respiratory depression	Hypotension
A	40	13.3	46.7	0	0
B	16.7^a^	23.3	26.7	0	0
C	10^a^	30	6.7^ab^	0	0

Data presented as %; *n* = 30 for all groups

^a^*P* < 0.05 versus group A; ^b^*P* < 0.05 versus group B

Airway resistance, the pressure difference created by the unit flow in the airway, is affected by the velocity of the airflow, the form of the airflow, and the diameter of the airway. With constant airflow velocity and airflow form, the size of the airway diameter is the most important factor that affects the airway resistance. Airway pressure is an important parameter for intraoperative monitoring of airway resistance. Excessive airway resistance can cause ventilator-associated lung injury [24]. Laparoscopic surgery is characterized by short surgical duration, minimal trauma, and rapid recovery; however, pneumoperitoneum may cause unpredictable changes in respiratory function [25]. In our study, we assessed the effect of the replacement of a single Murphy eye with overlaid microholes on the airway pressure. The results showed no significant difference between the three groups with respect to airway pressure either before, during, or after pneumoperitoneum (all *P* > 0.05) (Table 5). This suggests that the design change provided safe and effective improvement in ventilation.

**Table 5:** Comparison of airway resistance before, during, and after pneumoperitoneum in the three groups

Characteristics	Group			*F* value	*P*-value
	A (*n* = 30)	B (*n* = 30)	C (*n* = 30)		
Airway resistance before pneumoperitoneum (cm H_2_O)	12.4 ± 2.4	13.4 ± 3.5	12.8 ± 3.1	0.880	0.419
Airway resistance in pneumoperitoneum (cm H_2_O)	18.5 ± 2.8	18.3 ± 3.3	17.9 ± 3.7	0.264	0.768
Airway resistance after pneumoperitoneum (cm H_2_O)	13.7 ± 2.2	14.7 ± 3.4	14.5 ± 3.5	0.963	0.386

Data presented as mean ± standard deviation

Even with these promising findings, the limitations associated with the study design as well as the new intratracheal catheters should be clearly addressed. First of all, our study had a modest sample size. Further large scale study is required to provide more robust evidence. In addition, although we reduced the tube length by replacing the single Murphy eye with a group of miniholes, the function of Murphy eye as an alternative port of ventilation is also lost. Therefore, application of this modified tube, especially in patients with productive cough, may potentially compromise patient safety owing to inadequate ventilation. The suitable population for this tube should be carefully selected and further study is warranted to verify its safety profile. Last but not the least, although the new tube may depress the stress response associated with introduction of intubation and pre-extubation, it cannot blunt the sympathetic response to direct laryngoscopy.

In summary, the modified intratracheal catheters for drug delivery reduce cardiovascular and stress response during tracheal intubation and extubation in patients undergoing laparoscopic cholecystectomy, without increasing airway pressure. Based on the promising results of this single-center study, it is expected to apply such a device to more patients in more diverse clinical scenarios.

## Conclusion

The tested endotracheal anesthesia tube is effective to reduce cardiovascular and tracheal stress response and increase patient comfort, without inducing an increase in airway resistance.

## Data Availability

The data that support the findings of this study are available from the corresponding author upon reasonable request.

## Abbreviations

ASA: American Society of Anesthesiologists
DBP: Diastolic blood pressure
HR: Heart rate
I:E: Inspiratory: expiratory
ICU: Intensive care unit
NYHA: New York Heart Association
PEEP: Positive end-expiratory pressure
PetCO2: End-expiratory carbon dioxide partial pressure
SBP: Systolic blood pressure
